# Abdominal Pannus Delays Detection of Abdominal Wall Abscess

**DOI:** 10.7759/cureus.117732

**Published:** 2026-10-10

**Authors:** Connor Stauder, Zachary Cooper, Nicole M Lopez, Kelsey R Wilson

**Affiliations:** 1 Internal Medicine, Wright State Internal Medicine Residency, Dayton, USA; 2 Internal Medicine, Uniformed Services University of the Health Sciences, Bethesda, USA; 3 Internal Medicine, Keesler Medical Center, Keesler Air Force Base, USA; 4 Internal Medicine, Wright-Patterson Medical Center, Wright-Patterson Air Force Base, USA

**Keywords:** aeromonas hydrophila, bacteroides fragilis, multidisciplinary treatment, native mitral valve endocarditis, obesity-related illnesses, occult infection, primary abdominal wall abscess, streptococcus mitis

## Abstract

Abdominal wall abscesses (AWA) of primary etiology are rare, typically presenting in the context of atypical infections or in the setting of immunocompromise. Development of such pathology typically occurs only in the context of subclinical infections or severe systemic or metabolic disease. AWA more frequently develops as a secondary process due to underlying abdominal pathology such as chronic inflammatory conditions. In non-obese patients, AWA is often more readily apparent on exam with abnormalities of the abdominal contour, lending to prompt diagnosis and treatment, and even in those who are overweight, symptoms typically precede diagnosis as imaging is sought to delineate etiology.

Herein, we present the case of an 82-year-old, morbidly obese man with a known history of atrial fibrillation who presented for three days of mild pain and drainage from a purported spider bite to the right lower quadrant of the abdomen, located along a distended pannus that had itself reportedly been present for 10 years. Initial examination showed firmness and swelling of the pannus and purulent drainage with minimal erythema surrounding a <1 cm lesion. Labs demonstrated leukocytosis with a WBC of 19.2 x 10^3^/μL and subsequent computed tomography (CT) of the abdomen and pelvis showed a complex fluid collection in the subcutaneous soft tissue of the abdominal wall, 36.5 x 11.1 x 18.3 cm, consistent with AWA. Infectious management was initiated with broad-spectrum antibiotics, and he was taken for incision and drainage (I&D) once medically stable, with intraoperative findings confirming the AWA with surrounding rind, which was excised with subsequent washout and wound vac placement. The patient's clinical course was further complicated by recurrence of atrial fibrillation with BPM in the 110-130s range and subsequent discovery of a 5 mm mitral valve vegetation on transesophageal echocardiogram. Wound cultures grew *Bacteroides fragilis* and *Aeromonas hydrophila* while blood cultures, in contrast, grew *Streptococcus mitis*. With treatment of both his AWA and newly discovered mitral valve endocarditis, the patient improved and was eventually discharged from the hospital following confirmation of persistently negative blood cultures.

Primary AWA is a rare condition, and this report highlights a case which likely avoided detection for multiple years due to the patient's obese body habitus. The case strengthens the convention of treatment for primary AWA with combined surgical and antibiotic interventions and also highlights the need for extensive culture data to best characterize sources of infection in remote bodily sites. Overall, this helps to not only delineate treatment methods for a primary process, but to emphasize the importance of thorough, guided workup of an infectious process.

## Introduction

Abdominal wall abscesses (AWA) typically occur as a secondary process related to intra-abdominal infection, primary malignancy, surgical intervention, or inflammatory conditions of the gastrointestinal tract [1-5]. Spontaneously occurring primary AWAs, with no similar associated condition, are far less common. This largely owes to there being significant physiological separation between the abdominal cavity itself and the compartments of the intestine and surrounding organs that would allow seeding of microorganisms to such destinations [6]. Consequently, previous literature describes relatively few cases of primary AWA, with etiologies attributed to either unusual pathogens, such as tuberculosis [7] or actinomyces [8,9], or idiopathic in nature. Across many reviewed cases, abnormal abdominal contour caused by primary AWA is more readily apparent on exams due to the patients’ normal body habitus, leading to prompt diagnosis and treatment [7-14]. Despite this, few sources acknowledge bodily habitus as a possible factor impacting diagnosis. This case highlights such considerations as potentially clinically meaningful in avoiding delay or failure to diagnose this condition.

## Case presentation

An 82-year-old man with a history of type II diabetes mellitus, atrial fibrillation, and chronic heart failure with reduced ejection fraction presented to our hospital for a three-day history of mild pain and drainage in the right lower quadrant of the abdomen. The patient suspected this injury was secondary to a spider bite in the same area of the abdomen sustained on the day of symptom onset, noting no other recent injury, illness, surgery, or other insult that would have caused such a change. He reported being in otherwise good health and without other new concerns or symptoms.

On examination, the patient exhibited large body habitus (BMI 45.07 kg/m^2^) with firmness and swelling of the abdominal pannus. The patient reported that such findings had been present since an accident 10 years prior and did not feel this was unusual for him. Physical examination also demonstrated foul-smelling, purulent drainage and minimal erythema surrounding a <1 cm^2^ lesion in the right lower quadrant. Despite his other abdominal findings, the patient did not have any associated tenderness at the site of the lesion. The patient was initially afebrile with borderline hypotension, with a mean arterial pressure (MAP) of 70 mmHg, and in atrial fibrillation with a ventricular rate in the 110-130 bpm range. Labs demonstrated leukocytosis to 19.2 x 10^3^/μL (normal range 4.6-9.4 x 103/μL) with a neutrophilic predominance and creatinine elevated to 2.0 mg/dL from his baseline of 1.3 mg/dL (normal range 0.66-1.25 mg/dL). All other lab results were within the reference range.

A contrast-enhanced CT of the abdomen and pelvis showed a large, complex fluid collection in the subcutaneous soft tissue of the ventral infraumbilical abdominal wall measuring 36.5 x 11.1 x 18.3 cm, consistent with an abdominal wall abscess (Figure 1). The patient was started on ampicillin/sulbactam and linezolid and taken to the operating room (OR) for incision and drainage. Intraoperative findings confirmed a large abdominal wall abscess with a surrounding rind, densely adherent to the underlying fascia, which was excised.

**Figure 1:**
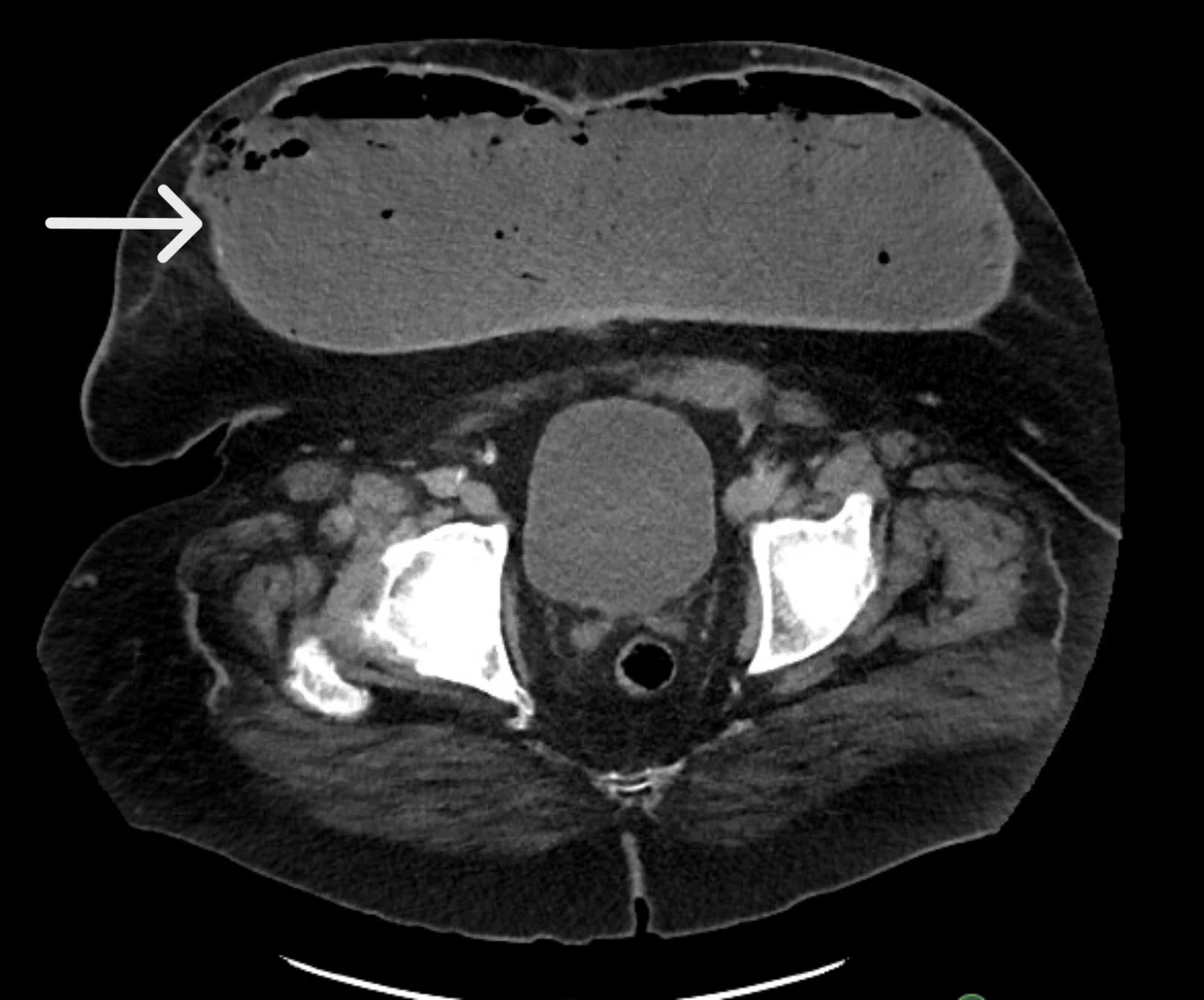
Contrast-enhanced CT scan of the abdomen showing large abdominal wall abscess measuring 36.5 x 11.1 x 18.3 cm White arrow indicates the area of abscess in the ventral abdominal wall

Though initially hemodynamically stable post-operatively, he subsequently developed ventricular rates up to the 150s bpm. Metoprolol was given to treat this. Subsequently, he became hypotensive with MAP of 50 mmHg, requiring support with norepinephrine. He was taken back to the OR the following day for repeat washout and wound vacuum placement and was subsequently able to come off norepinephrine. Blood cultures from admission grew *Streptococcus mitis*/*oralis* group, and abscess cultures grew *Bacteroides fragilis* and *Aeromonas hydrophila*. Considering these discordant findings, a transesophageal echocardiogram was performed, demonstrating a 5 mm mitral valve vegetation concerning for endocarditis of unclear chronicity (Figure 2).

**Figure 2:**
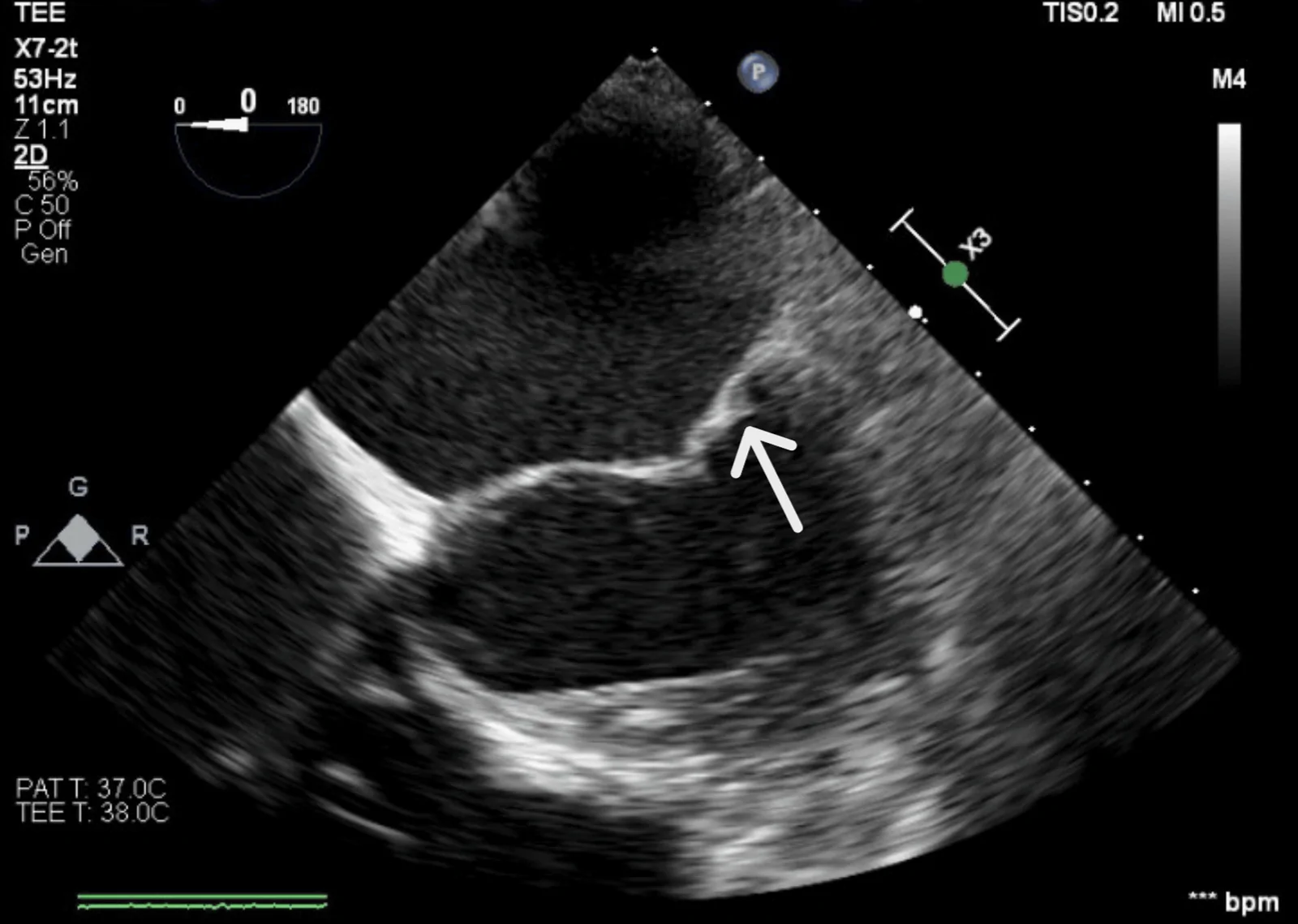
Transesophageal Echocardiogram demonstrating 0.5 cm vegetation adherent to the posterior mitral leaflet Arrow indicating adherent mass suspected to be posterior mitral valve leaflet vegetation

Infectious disease was consulted given the patient’s multifocal, polymicrobial, and high-risk infections, and antibiotic coverage was subsequently narrowed to ceftriaxone and metronidazole. After multiple days of treatment, subsequent blood cultures were persistently negative, and his leukocytosis and creatinine resolved. The patient was eventually discharged to a long-term acute care facility with continued IV antibiotics for infectious endocarditis, with a plan in place for outpatient infectious disease follow-up.

## Discussion

Amidst the relatively small grouping of reported cases of primary AWA described in the literature, many choose to focus on the causative organism or pathology that led to the development of such a condition. Existing data continue to support the notion that inflammatory disease, abdominal procedures, and immunocompromise all represent major risk factors, as exemplified by case reports authored by Neufeld et al. [2], Eisenstat [3], and Tamura et al. [13]. In addition, literature reviews such as that done by Shiihara et al. help to characterize the broad range of infectious organisms that can be found in these cases [10]. Collectively, these reports support the notion that diagnosing primary AWA requires a comprehensive diagnostic workup and thorough understanding of relevant risk factors.

This case not only bolsters the existing body of evidence for this disease process, but it also elaborates on a persistent concern raised in several prior articles: diagnostic delay. As described in a case by Panchagnula et al., the non-specific nature of the presenting symptoms and physical exam of primary AWA can create significant delay in the initial diagnosis and subsequent treatment, potentially even leading to mortality in such instances [1]. Several articles acknowledge this issue, particularly regarding the discordance between exam findings and extent of underlying disease, with some publications such as that of Mahajan et al. pointing out that symptoms can at times be minimal to nonexistent depending on the patient in question [11]. Such is the case with the patient presented here, with such discordance between his disease and minimal examination findings potentially attributable to his obese body habitus.

We thus propose that, within the context of the other diagnostic difficulties for primary AWA, other confounding factors including obesity must be considered as well. Of the articles mentioned above, none address this possibility as a potential barrier to initial diagnosis, though many of them recognize that a readily apparent and palpable mass on exam tends to make the diagnosis easier. In a process that frequently warrants both surgical and medical management, being aware of such potential confounders is therefore essential to ensuring prompt diagnosis and treatment.

## Conclusions

Despite a growing body of evidence, primary abdominal wall abscess remains a rarely reported and poorly understood condition. Among its many diagnostic barriers are its non-specific symptoms and minimal exam findings, both of which can argue in favor of a more conservative approach to imaging and treatment than the condition warrants. This diagnostic challenge may be further compounded by potential confounding factors such as obesity, which may contribute to disproportionately mild clinical presentations. This case highlights the importance of maintaining a high index of suspicion and pursuing appropriate imaging when clinical findings are discordant with the suspected diagnosis. Further study is needed to better characterize populations at risk for delayed diagnosis and underdiagnosis of primary abdominal wall abscess.
